# Combined subthalamic and nucleus basalis of Meynert deep brain stimulation for Parkinson’s disease with dementia (DEMPARK-DBS): protocol of a randomized, sham-controlled trial

**DOI:** 10.1186/s42466-020-00086-w

**Published:** 2020-10-19

**Authors:** Christine Daniels, Frank Steigerwald, Philipp Capetian, Cordula Matthies, Uwe Malzahn, Peter U. Heuschmann, Jens Volkmann

**Affiliations:** 1 Department of Neurology, University Hospital Würzburg, Josef-Schneider-Str. 11, 97080 Würzburg, Germany; 2 Department of Neurosurgery, University Hospital Würzburg, Josef-Schneider-Str. 11, 97080 Würzburg, Germany; 3 Clinical Trial Center, University Hospital Würzburg, Josef-Schneider-Str. 2, 97080 Würzburg, Germany; 4 Institute of Clinical Epidemiology and Biometry, University of Würzburg, Josef-Schneider-Str. 2, 97080 Würzburg, Germany

**Keywords:** Deep brain stimulation, Nucleus basalis of Meynert, Parkinson’s disease, Parkinson’s disease dementia, Subthalamic nucleus

## Abstract

**Introduction:**

Dementia in Parkinson’s disease (PDD) is a common non-motor symptom of advanced disease, associated with pronounced neocortical cholinergic deficits due to neurodegeneration of the nucleus basalis of Meynert (NBM) and its cholinergic terminals. In advanced PD, patients often require advanced therapies such as infusion therapy or deep brain stimulation (DBS) to improve motor control. However, patients with associated dementia are commonly excluded from DBS because of potential deterioration of cognitive functions. Yet marked reductions in dopaminergic medication and the subsequent risk of side effects (e.g., cognitive decline, psychosis, delirium) suggest that critical re-consideration of DBS of the subthalamic nucleus (STN-DBS) for advanced stages of PD and PDD is worthwhile. In this Phase 1b study, we will provide STN-DBS to a cohort of PDD patients with severe motor fluctuations and combine two additional electrodes for augmentative neurostimulation of the NBM.

**Methods:**

We aim to include 12 patients with mild-to-moderately severe PDD who fulfill indication criteria regarding motor symptoms for STN-DBS. Eligible patients will undergo implantation of a neurostimulation system with bilateral electrodes in both the STN and NBM. After 12 weeks of STN-DBS (visit 1/V1), participants will be randomized to receive either effective neurostimulation of the NBM (group 1) or sham stimulation of the NBM (group 2). NBM-DBS will be activated in all participants after 24 weeks of blinded treatment (visit 2/V2). The primary outcome will be the safety of combined bilateral STN- and NBM-DBS, determined by spontaneously-reported adverse events. Other outcome measures will comprise changes on scales evaluating cognition, activities of daily living functioning and clinical global impression, as well as motor functions, mood, behavior, caregiver burden and health economic aspects, and several domain-specific cognitive tests. Changes in scores (V1 – V2) for both treatment arms will undergo analysis of covariances, with baseline scores as covariates.

**Perspective:**

The feasibility and safety of combined STN-NBM-DBS in patients with PDD will be assessed to determine whether additional NBM-DBS improves or slows the progression of cognitive decline. Positive results would provide a basic concept for future studies evaluating the efficacy of NBM-DBS in larger PDD cohorts. Indirectly, proof-of-safety of STN-DBS in PDD might influence patient selection for this standard treatment option in advanced PD.

**Trial registration:**

ClinicalTrials.gov identifier (NCT number): NCT02589925.

## Introduction

Cognitive impairment is a common non-motor symptom of Parkinson’s disease (PD), and time of onset, severity, and cognitive profile show considerable interindividual variability [22]. Prevalence is high, with 80% of PD patients reported to experience dementia after 8 years [1]. Diagnosis of PD dementia (PDD) requires identification of cognitive deficits in at least two of the four core domains (attention, memory, executive and visuo-spatial functions) and consecutive impairment of normal functioning in everyday life [13].

Based on longitudinal neuropsychological examinations, Robbins and collaborators suggested a dual cognitive syndrome in PD: frontal-executive impairment and posterior cortical impairment [35, 40]. Frontal-executive functions (including flexibility, planning, switching between well-learned tasks, response inhibition, and working memory) may be impaired early in PD [10], and show some dopamine dependency [25]. Degeneration of the nigrostriatal and mesocorticolimbic dopamine pathways is supposed to be major pathophysiological correlate [25]. However, in some PD patients temporo-parietal cortical dysfunction (including impairment of visuospatial skills, semantic verbal fluency, auditory verbal learning, semantic and visual memory) complement or predominate the cognitive profile [2]. In non-demented patients with minimal cognitive impairment (MCI), visuospatial dysfunction is associated with a high risk of subsequent dementia [39]. Pagonabarraga et al. systematically evaluated a new neuropsychological test battery, with items sensitive for either frontal-executive or posterior cortical functions, and found that “cortical-type” item scores selectively helped to discriminate between demented and non-demented PD patients [32].

Progressive loss of cholinergic output from the nucleus basalis of Meynert (NBM) to neocortical regions may contribute to posterior cortical cognitive impairment [22]. In addition to degeneration of the dopaminergic system, severe loss of cholinergic neurons in the NBM is a consistent finding in neuropathological studies, and has been reported to differentiate PDD from non-demented PD patients [17]. Hall et al. performed an elaborate histopathological analysis of the basal forebrain cholinergic nuclei of post-mortem brain tissue in PD and PDD patients and healthy controls. They found: 1) a frank loss of NBM neurons in PDD, 2) α-synuclein deposition in the NBM that was significantly higher in PDD versus PD, 3) a decrease in cholineacetyltransferase (CAT) activity as a marker of cholinergic transmission in the hippocampus in PDD, and 4) frontal neocortical loss of CAT activity in PD and PDD. Cholinergic dysfunction of the frontal cortex is also present in non-demented PD patients without significant structural loss of NBM neurons, and has been attributed to α-synuclein deposition in NBM neurons that may decrease neurotransmitter production [21]. The NBM sends out projections to medial temporal structures, the amygdala, the frontoparietal cortex, and temporoparietal association areas [18]. It is the major source of cholinergic transmission to the neocortex, and has been functionally associated with the control of attention and maintenance of arousal, both key functions for appropriate learning and memory formation [5]. Molecular imaging of acetylcholinesterase activity by positron emission tomography (PET) revealed that cholinergic denervation of the cerebral cortex is an early phenomenon in PD and is more widespread and profound in PDD patients [37].

Recently, advanced MRI-based morphometric analysis techniques of the NBM volume have been developed [23], and loss of NBM volume in PD is associated with baseline cognitive deficits [7, 16] and longitudinal cognitive decline [34, 36]. Recently, Pereira et al. [33] performed a longitudinal study in patients with PDD or who developed PDD during the study. They reported that atrophy of the NBM precedes and predicts future dementia in PD. Baseline volumes and longitudinal changes in the NBM were associated with worse global cognition and semantic and phonemic fluency abilities. In accordance with the functional deficit of posterior cortical regions, Gang et al. showed an association between reductions in NBM volume and parietal glucose metabolism in PDD [15].

Rivastigmine is the only approved medication addressing cognitive impairment in PDD, and effect sizes in clinical studies were relatively small [3]. No disease-modifying treatments with effects on cognition in PD are currently available. Deep brain stimulation (DBS) is an established, highly-effective treatment option in movement disorders [29], with an evolving spectrum of indications that increasingly includes psychiatric disorders [28]. DBS of the NBM has been used in single cases and small patient cohorts. In contrast to conventional DBS targets, NBM-DBS has some exceptional features as the degenerating nucleus itself is the target for electrode placement [26], requiring low-frequency stimulation to activate axon structures. Low-frequency NBM stimulation in rats led to a marked increase in acetylcholine emission in the neocortex [27]. Comparable animal trials demonstrated amelioration of attention and memory functions after NBM-DBS, and more impaired baseline memory functions led to greater effects [9, 24, 30].

A first case study published by Freund et al. reported the effects of bilateral NBM-DBS and subthalamic nucleus (STN)-DBS as a standard treatment of motor symptoms in a 71-year-old man with severe PDD [14]. Improvements in short-term memory, attention, concentration, alertness, drive, spontaneity, and preoperative apraxia were reported following NBM-DBS (20 Hz), which remained stable over 18 months [6]. Gratwicke et al. [19] recently published an exploratory randomized, double-blind, crossover trial of NBM-DBS in six patients with PDD. Electrodes were placed on a trajectory that straddled the internal part of the globus pallidus internus (GPI), providing the potential for subsequent conventional GPI-DBS for coexisting motor impairments. After surgery, patients were assigned to receive active NBM-DBS or sham stimulation for 6 weeks, with crossover for another 6 weeks. The primary outcome was the difference in scores measuring verbal learning, working memory, verbal fluency, attention, and psychomotor speed/reaction times between groups. Surgery and stimulation were well tolerated by all six patients, with no reported serious adverse events. No consistent improvements were observed in primary cognitive outcomes or the resting-state functional magnetic resonance imaging. However, an improvement in Neuropsychiatric Inventory (NPI) scores was observed in two patients with NBM-DBS.

Case reports and small exploratory trials applying NBM-DBS in PD-associated MCI, Alzheimer’s disease (AD), and dementia with Lewy bodies (DLB) have also been reported. Nombela et al. described NBM-DBS in a patient with PD-associated MCI [31]. NBM-DBS was combined with GPI-DBS via the same lead, using different contacts and different stimulation frequencies. After 3 months of combined GPI/NBM-DBS, improvements were noted in all neuropsychological measurements apart from semantic verbal fluency and reverse digit span. In the AD trial, cognition (Mini-Mental State Examination, MMSE) was stable over 12 months after DBS in all 10 patients and correlated with a preserved fronto-parieto-temporal cortical thickness [4]. Gratwicke et al. replicated their randomized, double-blind, crossover trial in six patients with DLB [20]. No consistent improvements were observed in exploratory clinical outcome measures, but the severity of neuropsychiatric symptoms reduced with NBM-DBS in three patients. In all published cases, NBM-DBS proved to be feasible and well tolerated, with only temporary related adverse events.

Thus, initial results in PDD, AD, and DLB demonstrate the safety and feasibility of NBM-DBS and provide an indication of efficacy regarding neuropsychiatric symptoms. However, neurosurgical intervention for implantation of NBM electrodes alone is currently unjustifiable because it would not address the complex motor and non-motor syndrome associated with advanced PD. Therefore, in our study only patients fulfilling established motor indications (motor response fluctuations or dyskinesia) for STN-DBS will be selected [8]. Patients with cognitive impairment are commonly excluded from this therapy due to safety concerns. This exclusion, based on expert opinion and fear of perioperative delirium or cognitive worsening rather than controlled clinical trial results, needs to be critically reconsidered given the increasing evidence regarding the cognitive safety of STN-DBS in non-demented patients and improved surgical techniques. Moreover, STN-DBS allows a marked reduction in dopaminergic medication, now an indication for this therapy in cases of non-motor dopaminergic adverse events such as impulse control disorders or levodopa-dysregulation syndrome, which are difficult to manage pharmacologically [38]. The reduction in dopaminergic medication may also benefit PDD patients, to reduce the risk of delirium or hyperdopaminergic behaviors aggravating PDD-associated symptoms.

## Methods

### Aim of the trial

The only currently-available treatment option in PDD consists of oral rivastigmine, which increases acetylcholine levels in the brain by inhibiting cholinesterase [12]. Its efficacy is limited, especially when compared to the effects of levodopa on motor symptoms. Disease-modifying treatment strategies are lacking [2]. Following the assumption of initial axonal dysfunction and subsequent neurodegeneration of the NBM, augmentative neurostimulation of the NBM should increase cholinergic output from the basal forebrain and reconstitute neocortical functions. Our study (DEMPARK-DBS) will evaluate the safety of combined bilateral STN-NBM-DBS in PDD patients, and will clarify whether additional NBM-DBS improves or slows cognitive decline.

### Study description and study design

**Fig. 1:**
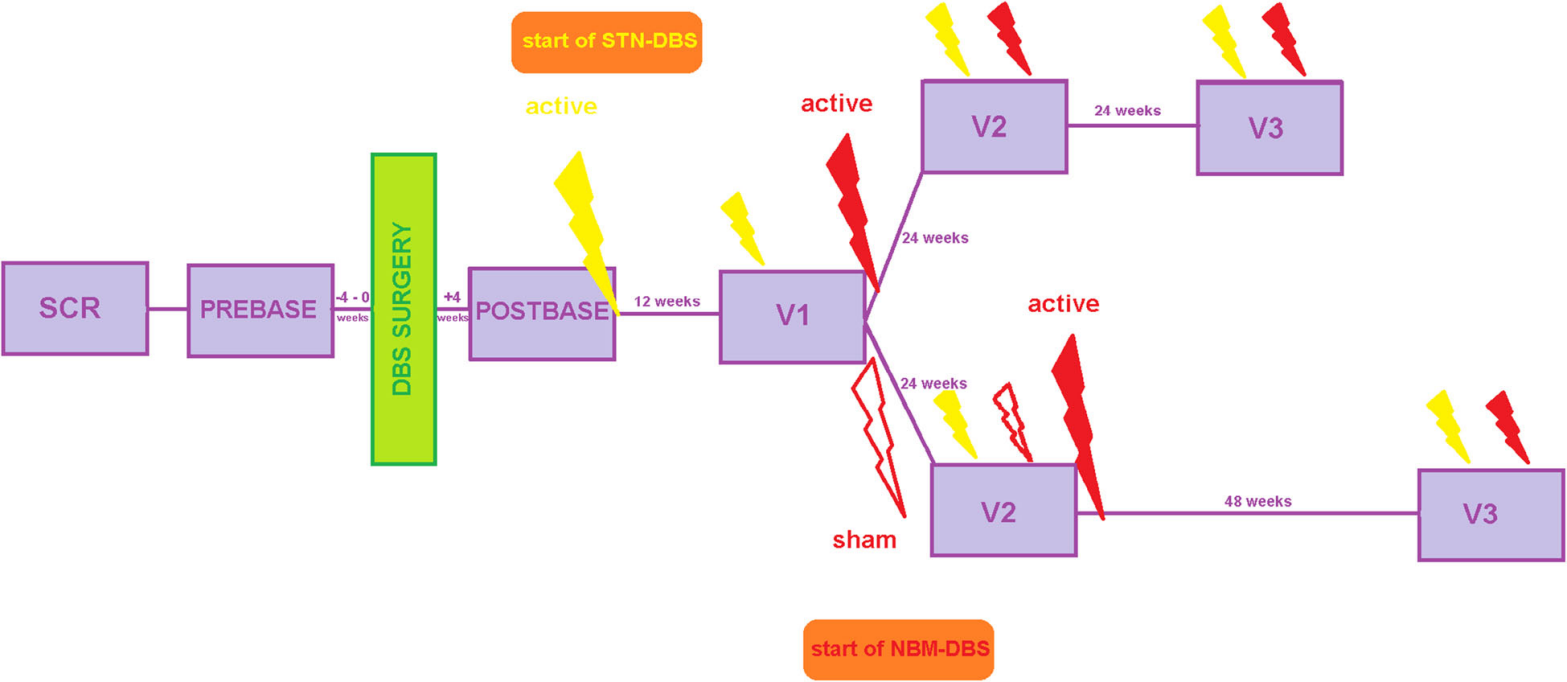
Visit schedule of DEMPARK-DBS: Icon “flash” = ‘activation’ or ‘active’ DBS. PREBASE, presurgical baseline visit; POSTBASE, postsurgical baseline visit; SCR, screening visit; V1, Visit 1; V2, Visit 2; V3, Visit 3

This will be a prospective, single center, Phase 1b study with a double-blind, randomized, sham-stimulation controlled, delayed activation of NBM-DBS (staggered onset) design. We aim to include 12 patients with PDD fulfilling eligibility criteria listed in Table 1. Patients must be on stable doses of antiparkinsonian and antidementia medications for at least 4 weeks prior to screening assessment. Medications and dosages may be adjusted as necessary after DBS surgery. Figure 1 shows the study schedule of DEMPARK-DBS.

**Table 1:** Eligibility criteria

Inclusion criteria:	Exclusion criteria:
1. Age at enrollment: 35–75 years. 2. Diagnosis of idiopathic PD with probable PDD, defined by MDS consensus guidelines [13]. 3. Mild-to-moderately severe dementia, defined by MMSE score 10–24. 4. Duration of bilateral idiopathic PD: ≥5 years of motor symptoms. 5. Severity of bilateral idiopathic PD in medication-off state: modified Hoehn and Yahr stage ≥2. 6. Unified Parkinson’s Disease Rating Scale (UPDRS) III score ≥ 30 in medication-off/stimulation-off state. 7. Levodopa must improve PD symptoms by ≥30% in levodopa challenge test (UPDRS III score). 8. PDD with symptom onset at least 2 years after first symptoms of PD. 9. Willing and able to comply with all visits and study-related procedures (e.g., using the remote control and charging systems, completing the motor diary) if mentally competent or, if incompetent, their legally-authorized representatives. 10. Able to understand the study requirements and treatment procedures and provide written informed consent before any study-specific tests or procedures are performed. If mentally incompetent, the legally-authorized representative will provide written informed consent.	1. Any significant psychiatric problems, including acute confusional state (delirium), ongoing psychosis, or clinically significant depression. 2. Any current drug or alcohol abuse. 3. Any history of recurrent or unprovoked seizures. 4. Any prior movement disorder treatments involving intracranial surgery or device implantation. 5. History of neurostimulation intolerance in any area of the body. 6. Any significant medical condition likely to interfere with study procedures or confound evaluation of study endpoints, including any terminal illness with survival < 12 months. 7. Participation in another drug, device, or biologics trial concurrently or within the preceding 30 days. Any other trial participation should be approved by the Principal Investigators. 8. Pregnancy, breastfeeding, or lack of reliable contraception.

During the screening visit, the PDD diagnosis will be confirmed by the Movement Disorders Society (MDS) criteria using the algorithm for PDD at level I [11]. The MMSE total score must lie between 10 and 24 points, indicating mild-to-moderately severe dementia. Patients will fulfill clinical inclusion criteria for STN-DBS in terms of motor scores and requirements for local/general anesthesia.

The preoperative baseline examination will be performed 4 weeks prior to the DBS operation. Table 2 shows the full data set to be collected at pre- and postoperative Baseline and Visits 1–3 to achieve exploratory study endpoints, and includes assessment of motor function in the medication-“off” state after 12-h withdrawal of dopaminergic medication and medication-“on” state after standard levodopa challenge; video recording will also be used for documentation. All other scores and scales will be performed in the medication-“on” state, to assess global cognitive function, neuropsychiatric symptoms, and a number of domain-specific tests as part of a detailed neuropsychiatric battery, as well as quality of life, health economics, and caregiver burden. During all study visits, adverse events will be documented and rated for frequency and severity.

**Table 2:** Data set: assessment at presurgical/postsurgical baseline, visits 1–3

Motor assessments	• Movement Disorder Society-Unified Parkinson’s Disease Rating Scale (MDS-UPDRS) III (medication-off/−on state) • MDS-UPDRS I, IV • Clinical Dyskinesia Rating Scale (CDRS)
Global cognitive function	• Alzheimer’s Disease Assessment Scale, cognitive subscale (ADAS-cog)
Activities of daily living	• Alzheimer’s Disease Cooperative Study, Activities of Daily Living (ADCS-ADL) • MDS-UPDRS II
Neuropsychiatric symptoms	• Beck Depression Inventory (BDI) II, • Starkstein Apathy Scale • Neuropsychiatric inventory (NPI)
Domain specific (frontal executive) tests	• Delis-Kaplan Executive Function Systems (D-KEFS) verbal fluency battery • Wisconsin Card Sorting Test (modified version) • Trail Making Task (TMT) Part A + B • Stroop Test (Victoria Version) • Symbol Digit Modalities Test (SDMT)
Domain specific (attention) test	• Brief Test of Attention (BTA)
Quality of life	• Parkinson’s Disease Questionnaire (PDQ39) • EuroQol (EQ)-5d
Health economics	• EQ-5d/quality-adjusted life years (QALYs)
Caregiver burden/quality of life	• Zarit Burden Interview (ZBI) • Short form (SF)-36
Clinical global impression of change	• Baseline interview: Visit 1 • Follow-up: Visits 2 & 3

All participants will undergo stereotactic implantation of deep brain electrodes. The neurostimulation device used will be the Vercise™ System (Boston Scientific Corporation (BSC)), consisting of an implantable pulse generator (IPG), integrated rechargeable battery, DBS leads, surgical tools, and external devices (programming system, remote control, and charging system; Table 3). To allow delivery of stimulation pulses to four DBS electrodes with different stimulation frequencies (STN: 100–200 Hz; NBM: 20–80 Hz), the 22-cc Precision Spinal Cord Stimulation IPG II splitter (BSC) will be used. The DBS procedure will follow local standard operating procedures on perioperative management and stereotactic procedure. A preoperative MRI-scan under general anesthesia and postoperative image control of electrode positioning will be performed in all patients.

**Table 3:** Details of the vercise™ neuromodulation system (Boston Scientific Corporation (BSC))

Vercise TM IPG Kit Model Nr. DB-1110-C	
Vercise TM Lead Kit, 30 cm Model Nr. DB-2201-30-C	
55 cm 8 Contact Extension Kit Model Nr. NM-3138-55	
Vercise TM Physician’s Spare Kit Model Nr. DB-2500-C	
Vercise TM Clinician Programmer (M400) Model Nr. DB-7151-20-C	
Tunneling Tool, 35 cm Long Model Nr. SC-4254	
Holder, IR Interface Model Nr. NM-4502	
Vercise TM Charging Collar Aceesseries Model Nr. DB-6300-C	
Vercise TM Remote Control Kit Model Nr. DB-5500-C	
Vercise TM Remote Control w/batteries (Ti) Model Nr. DB-5212-C	
Vercise TM Charging System Kit Model Nr. DB-6412-EU-C	
Charger Model Nr. NM-5312	
Base Station Model Nr. NM-5305	
D4 Splitter 2 × 4 Model Nr. SC-3304-xx	

Postsurgical baseline evaluation will be performed at 4(±1) weeks after surgery. Motor assessments will be performed in the medication-“off” state. Subthalamic neurostimulation will be initiated in all patients using individualized stimulation parameters determined via monopolar review.

Twelve weeks after STN activation, neurostimulation Visit 1 (V1) will take place. At this time, baseline evaluation of the clinical global impression of change (ADCS-CGIC) will be performed by a blinded rater not involved in medical treatment and with no information about the results of other scales and scores. For the first baseline interview with the patient and caregiver, documented medical history will be the only additional information that may be studied. Motor assessments will be performed in the medication-“off” and -“on” state with active STN-DBS; all other scales and scores will be evaluated in the medication-“on” state with active STN-DBS. Subsequently, medication and STN-DBS parameters will be optimized if necessary, followed by 1:1 blinded randomization to active or sham NBM-DBS. After another 24 weeks, Visit 2 (V2) will take place. To evaluate CGIC compared to V1, patient and caregivers will be re-interviewed by the blinded rater that performed the baseline interview. This time, no additional information may be provided from medical records or other information sources. Motor assessments will be performed in the medication-“off” and -“on” state with active STN-DBS and active or sham NBM-DBS; all other scales and scores will be evaluated in the medication-“on” state with active STN-DBS and active or sham NBM-DBS. Subsequently, STN-DBS will be adapted if necessary and active NBM-DBS continued in all patients. Visit 3 (V3) will be performed 48 weeks after activation of NBM-DBS in both patient groups, i.e., 24 weeks after V2 in the NBM-DBS group, 48 weeks after V2 in the sham NBM-DBS group. The CGIC will again be rated compared to V1. After V3, the study will end and medication, STN-DBS, and NBM-DBS will be adjusted without restrictions according to clinical needs. An annual follow-up visit for up to 5 years after activation of NBM-DBS will be provided to all participants.

### Arms and interventions

Eligible patients who consent to participation and meet all inclusion and exclusion criteria will receive the following settings in a pre-specified randomized order for NBM-DBS at V2: test stimulation at 60 μs, 20 Hz, and individually-adjusted amplitude (verified using the GuideXT visualization tool (BSC) if necessary) below the threshold of adverse effects; sham stimulation at 0 V, 60 μs, 20 Hz. As we do not anticipate any specific clinical effects after NBM-DBS activation, blinding will be secured by documenting NBM-DBS parameters independent from medical records. Randomization and programming of NMB-DBS will be restricted to three members of the study team not involved in other diagnostic procedures.

### Outcome measures

The primary endpoint will be the safety of combined STN-NBM-DBS, determined by spontaneously-reported adverse events. Exploratory endpoints will comprise the change between V2 to V1 concerning the scales and scores of the full data set listed in Table 2.

### Statistical methods

This study is exploratory to provide necessary data for sample size considerations for a possible subsequent pivotal trial. Sample size considerations are based on research by Emre et al. [12]; they found a 2.1 ± 8.2 point improvement on ADAS-cog with rivastigmine treatment compared to a worsening of 0.7 ± 7.5 points with placebo after 24 weeks (baseline 23.8 ± 10.2 points). Clearly, such small mean differences in combination with comparatively large standard deviations shown by the rivastigmine study will be only be detectable with appropriate power with sample sizes (2*125 = 250) far above that planned for our study. However, we hope to encounter considerably larger effects for NBM-DBS. Within this purely explorative analysis, we will test the null hypothesis of equal mean ADAS-cog change scores from V1 to V2 for both study treatments (STN-DBS + NBM-DBS versus STN-DBS + sham NBM-DBS) by a Type III F-test within an analysis of covariance (ANCOVA), with baseline ADAS-cog as covariate. Assuming a small-to-moderate correlation between baseline ADAS-cog and ADAS-cog change score from V1 to V2, a sample size of 2*6 = 12 patients ensures a power of 80% to detect a standardized mean difference of 1.66 as significant deviation from the null hypothesis of equal mean change scores for both treatments at significance level 0.05. This means we will be able to detect only large effects as significant, but that is not the main objective of this pilot study.

Note that because of the small sample size, statistical testing will not be the focus of data analysis. Despite the known robustness of ANCOVA, it may be difficult to detect violations in ANCOVA assumptions. A critical issue comes from possible differences in the sample distributions of baseline variables between the two treatment groups when comparing mean change scores for endpoints. If appropriate mean change scores resp. distributions of change score will be compared without adjustment for baseline variables by the two-sided T-test resp. by the Mann-Whitney test instead of an F-test within ANCOVA.

### Perspective

DEMPARK-DBS will be the first controlled study to evaluate combined STN-NBM-DBS using four electrodes as originally described by Freund et al. [14]. We will assess the feasibility and safety of combined STN-NBM-DBS in patients with PDD and will determine whether additional NBM-DBS improves or slows the progression of cognitive decline. Positive results would provide a basic concept for future studies evaluating the efficacy of NBM-DBS in larger PDD cohorts.

Dopaminergic treatment may worsen cognitive functions in advanced PD [25], and has a number of side effects that amplify behavioral disorders also primarily caused by dementia. STN-DBS allows a significant reduction in dopaminergic medication; by reducing associated hyperdopaminergic cognitive and behavioral disorders, it could benefit PDD patients. Proof-of-safety of STN-DBS in PDD might also have an impact on the general selection criteria for DBS in patients with advanced PD.

## Data Availability

Not applicable.

## Abbreviations

Ach: Acetylcholine
AChE: Acetylcholinesterase
ADAS-cog: Alzheimer’s Disease Assessment Scale, cognitive subscale
ADCS-ADL: Alzheimer’s Disease Cooperative Study, Activities of Daily Living
ADCS-CGIC: Alzheimer’s Disease Cooperative Study, Clinical Global Impression of Change
ADL: Activities of daily living
BDI: Beck Depression Inventory
BSC: Boston Scientific Corporation
BTA: Brief Test of Attention
CAT: Cholineacetyltransferase
CDRS: Clinical Dyskinesia Rating Scale
DBS: Deep brain stimulation
D-KEFS: Delis-Kaplan Executive Function Systems
EQ-5d: EuroQol
MCI: Mild cognitive impairment
MDS: Movement Disorder Society
MMSE: Mini-Mental State Examination
NBM: Nucleus basalis of Meynert
NPI: Neuropsychiatric Inventory
PD: Parkinson’s disease
PDD: Parkinson’s disease dementia
PDQ: Parkinson’s Disease Questionnaire
PET: Positron Emission Tomography
QALY: Quality-adjusted life year
SDMT: Symbol Digit Modalities Test
SF: Short Form (SF-36)
STN: Subthalamic nucleus
TMT: Trail Making Task
UPDRS: Unified Parkinson’s Disease Rating Scale
ZBI: Zarit Burden Interview
